# Modulation of Endoplasmic Reticulum Stress via CEBPB: A Potential Molecular Link to Therapeutic Action in Substance Use Disorders

**DOI:** 10.1002/cns.71183

**Published:** 2026-09-29

**Authors:** Cheng Zhang, Hu Li, Ming‐Fen Ho

**Affiliations:** ^1^ Department of Molecular Pharmacology and Experimental Therapeutics Mayo Clinic Rochester Minnesota USA; ^2^ Department of Psychiatry and Psychology Mayo Clinic Rochester Minnesota USA

**Keywords:** brain organoids, ER stress, iPSC, opioid addiction, pharmacotherapy

## Abstract

**Aims:**

Opioid use disorder remains a major public health crisis in the United States, driven largely by rising overdose deaths from synthetic opioids, such as fentanyl. Emerging population‐level evidence suggests that GLP‐1 receptor agonists (GLP‐1RAs) may reduce overdose risk. However, their underlying molecular mechanisms are not well understood. We investigated the transcriptional effects of fentanyl and GLP‐1RAs using human induced pluripotent stem cell (iPSC)‐derived forebrain organoids and neurons.

**Methods:**

Organoids were treated with fentanyl, liraglutide, or exenatide, followed by RNA sequencing. We extended these analyses to iPSC‐derived neurons exposed to additional therapeutic candidates, including the anticonvulsants topiramate and gabapentin, and the metabolic modulator β‐hydroxybutyrate. All compounds were tested at clinically relevant concentrations, and transcriptomic and functional genomic assays were performed.

**Results:**

Across models and drug classes, we identified modulation of endoplasmic reticulum (ER) stress signaling as a shared molecular mechanism. Fentanyl and GLP‐1RAs consistently downregulated ER stress‐related genes, with TRIB3 as the most strongly suppressed target. We further identified CEBPB as a key upstream regulator and confirmed reduced CEBPB DNA‐binding activity in ER stress‐related genes.

**Conclusion:**

These findings highlight ER stress modulation as a convergent pathway with potential therapeutic relevance for substance use disorders.

## Graphical abstract

We identified endoplasmic reticulum (ER) stress modulation as a convergent transcription signature across fentanyl and multiple substance use disorder (SUD)‐relevant treatments. Key ER stress genes, including TRIB3, XBP1, and DDIT3, were downregulated, while CEBPB emerged as a shared upstream regulator linked to ER stress–related proteins. These findings suggest that suppression of ER stress signaling may represent a shared molecular response across SUD‐relevant treatments.

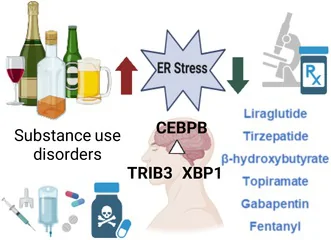

**Abbreviations**
AUD: alcohol use disorder
CDC: Centers for Disease Control and Prevention
ChIP: chromatin immunoprecipitation
co‐IP: co‐immunoprecipitation
DEGs: differentially expressed genes
EB: embryoid body
ER: endoplasmic reticulum
FDA: Food and Drug Administration
GIP: glucose‐dependent insulinotropic polypeptide
OUD: opioid use disorder
SUD: substance use disorders
T2D: type 2 diabetes

## 1 Introduction

In recent years, synthetic opioid‐involved overdose deaths have increased sharply [1]. More than 70,000 drug‐overdose deaths occurred in the US in 2019, and more than 50% involved synthetic opioids [1]. The misuse of prescription opioids, such as oxycodone, and illicitly manufactured fentanyl remains a major public health concern [2]. The US Centers for Disease Control and Prevention (CDC) indicates that the US is currently in a “third wave” of opioid overdose deaths driven primarily by synthetic opioids like fentanyl [3, 4, 5]. The age‐adjusted synthetic opioid death rate increased sharply by 1040% from 2013 to 2019 [1]. There are currently only three medications approved by the US Food and Drug Administration (FDA) for opioid use disorder (OUD): methadone, a full agonist of the μ opioid receptor; buprenorphine, a high‐affinity partial agonist of the μ and κ opioid receptors; and naltrexone, a μ‐opioid receptor antagonist [6]. Notably, the medications are underused. Only 1 in 5 US adults with OUD received medications to treat it in 2021 [7]. In contrast, type 2 diabetes (T2D), which is a prevalent medical condition worldwide, currently has over 37 FDA‐approved medications that comprise 10 drug classes available for treatment [8]. The overwhelming scale and cost of the opioid epidemic, along with the fact that there are relatively few medications for OUD, have increased the urgency to discover new therapeutic options.

GLP‐1 receptor agonists (GLP‐1RAs) such as semaglutide have been linked to a reduced risk of opioid overdose among patients with comorbid T2D and OUD [9]. Consistent with this finding, a recent study reported that individuals with OUD who received glucose‐dependent insulinotropic polypeptide (GIP)/GLP‐1 receptor agonist prescriptions experienced lower rates of opioid overdose compared with those who did not have these prescriptions [10]. Preclinical evidence further supports this potential relationship: a recent study demonstrated that exendin‐4 can cross the blood–brain barrier and attenuate opioid reinforcement and drug‐seeking behavior without impairing opioid‐induced analgesia in rats being treated with oxycodone [11]. Collectively, this body of work highlights the potential therapeutic value of GLP‐1 receptor agonists in OUD. Despite these observations, the mechanisms underlying this protective association remain unclear, prompting us to investigate the molecular signatures of fentanyl and GLP‐1 receptor agonists in induced pluripotent stem cell (iPSC)‐derived brain‐like cells.

A central challenge in neuropsychopharmacology is limited access to living human brain tissue, the primary site of pathology; this constraint limits our ability to explore mechanisms and identify therapeutic targets. Patient‐derived iPSC models help overcome this barrier by providing cells that retain individuals' genetic background and can be differentiated into human brain‐like tissue. This system also allows controlled experimental manipulation of living, brain‐relevant cells. Gaining insight into how opioids and GLP‐1RAs act at cellular and molecular levels may reveal new therapeutic targets for OUD.

The present study was designed to investigate the molecular mechanisms of fentanyl and GLP‐1 receptor agonists in iPSC‐derived brain organoids. Notably, we found that the most significant differentially expressed gene across all treatment conditions was tribbles pseudokinase 3 (TRIB3), a gene that is highly expressed in neurons and associated with endoplasmic reticulum (ER) stress. We then conducted functional genomics studies using iPSC‐derived forebrain neurons and discovered that CCAAT/enhancer‐binding protein beta (CEBPB) functions as a transcription factor that could alter the ER stress signaling pathway. Interestingly, in addition to GLP‐1 receptor agonists, several medications with potential therapeutic benefits for substance use disorders (SUDs) produced transcriptional signatures consistent with attenuation of ER stress signaling [12].

## 2 Materials and Methods

### 2.1 Generation of iPSCs, iPSC‐Derived Forebrain Organoids and Neurons

We generated three iPSC lines for brain organoid differentiation (2 male and 1 female participants) using peripheral blood mononuclear cells (PBMCs) and the CytoTune‐iPS 2.0 Sendai Reprogramming Kit (A16517, Thermo Fisher, USA) as described previously [13, 14]. All iPS cell lines showed normal karyotypes, and they all expressed pluripotency markers (Figure 1). Cell lines were regularly characterized and verified to be free from mycoplasma. We then generated iPSC‐derived forebrain organoids and neurons as reported previously [13, 15].

**FIGURE 1:**
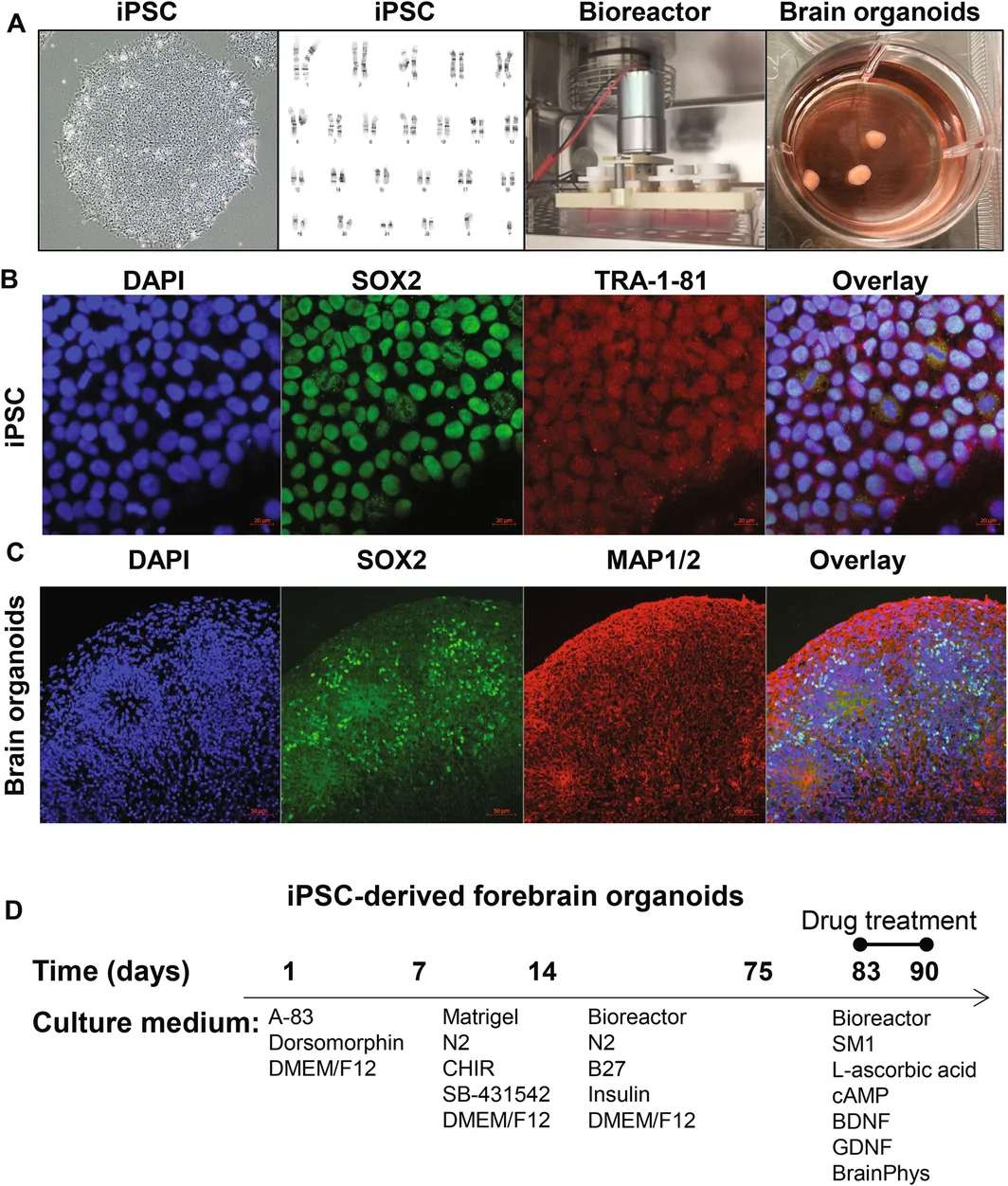
Generation and characterization of iPSC‐derived forebrain organoids. (A) Induced pluripotent stem cells (iPSCs) were cultured on Matrigel‐coated plates. G‐band karyotyping confirmed that all iPS cell lines used in this study had normal karyotypes. A bioreactor system was employed to grow iPSC‐derived brain organoids. (B) All iPSC lines expressed key pluripotency markers. Panel B shows representative immunostaining for the pluripotency markers SOX2 and TRA‐1‐81. (C) Panel C presents representative immunostaining images of day‐90 iPSC‐derived forebrain organoids, highlighting SOX2‐positive progenitor cells and MAP1/2‐positive neurons. (D) A schematic overview of the differentiation protocol used to generate the iPSC‐derived forebrain organoids.

3D iPSC‐derived forebrain organoids were generated from 3 individuals. Briefly, iPS cells were cultured on Matrigel with mTeSR1 Plus media (STEMCELL technologies, MA, USA). Embryoid bodies (EBs) were embedded in Matrigel and cultured with 1 × N2, 1 × NEAA, and 1 × Glutamax (Invitrogen, Grand Island, NY), 1 μM SB431542, and 1 μM CHIR99021 (Selleckchem) for a week. On Day 14, organoids were cultured in a bioreactor [16]. Culture medium from Days 14–70 consisted of DMEM/F12 medium supplemented with 1 × N2, 1 × B27, 1 × NEAA, and 1 × Glutamax, 1 × 2‐mercaptoethanol, 100 × penicillin–streptomycin solution, and 2.5 μg/mL insulin (Sigma‐Aldrich). The medium was changed every other day. From Day 70 onward, Brainphys medium supplemented with 20 ng/mL BDNF, 20 ng/mL GDNF (Peprotech), 0.2 mM L‐ascorbic acid, and 0.5 mM cAMP (Sigma‐Aldrich) was used [17, 18]. Drug treatment for iPSC‐derived forebrain organoids was initiated from Day 83 through Day 90 [15].

In addition, we generated iPSC‐derived forebrain neurons for follow‐up experiments. Neural progenitor cells were produced using the STEMdiff SMADi Neural Induction Kit (catalog #08581). These progenitors were then differentiated into a mixed population of forebrain‐type (FOXG1‐positive) neurons using the STEMdiff Forebrain Neuron Differentiation Kit in combination with the STEMdiff Forebrain Neuron Maturation Kit (catalog #08600). Maturation medium was replaced every 3 days during continuous culture [19]. The iPSC‐derived neurons were functionally mature by 4 weeks after differentiation from NPCs. These neurons formed synapses, fired action potentials, and had spontaneous synaptic activity [18, 19]. Drug treatment for iPSC‐derived forebrain neurons was initiated 4 weeks after differentiation.

### 2.2 Drug Treatment

Concentrations of fentanyl (3 nM) [20], liraglutide (50 nM) [21] and exenatide (150 pM) [22] were chosen to reflect blood drug levels observed in clinical studies. We treated iPSC‐derived forebrain organoids with vehicle, fentanyl, liraglutide, or exenatide for 7 days, with a daily medium change. Cells were collected for RNA extraction 24 h after the final treatment. A similar experiment was conducted in iPSC‐derived neurons that were treated with fentanyl (3 nM) [20], liraglutide (50 nM) [21], exenatide (150 pM), tirzepatide (100 nM) [23], topiramate (5 μM) [24], gabapentin (22 μM) [25], β‐hydroxybutyrate (5 mM) [26] or thapsigargin (100 nM).

### 2.3 Immunostaining and Confocal Microscopy

Cells were fixed with 4% paraformaldehyde at room temperature for 15 min, followed by permeabilization using 0.2% Triton X‐100 in PBS. Brain organoids were sectioned at a thickness of 10 μm. After blocking with 5% donkey serum in PBS for 30 min at room temperature, cells were incubated overnight with primary antibodies (see Table S1) diluted in 3% BSA. Following washes, secondary antibodies were applied for 1 h at room temperature. Nuclei were counterstained using antifade mounting medium containing DAPI (VECTOR Laboratories, Burlingame, CA, USA). Fluorescence images were acquired using an Olympus FV1200 confocal microscope.

### 2.4 RNA Sequencing (RNA‐Seq) and Data Analysis

Total RNA from iPSC‐derived brain organoids and neurons was extracted using Trizol and the RNeasy mini kit (Qiagen, Valencia, CA, USA). The RNA integrity numbers (RIN) were between 9.6 and 9.9 for all RNA samples. RNA‐seq in iPSC‐derived forebrain organoids included two technical replicates for sequencing (*n* = 4). RNA‐seq experiments were conducted by Azenta using an Illumina HiSeq 6000 with eight samples in each lane using 100 bp paired end index reads. Fastq files containing paired RNA‐Seq reads were aligned with STAR [27] against the UCSC human reference genome (GRCh38.p14). Approximately 30 million reads were generated per sample, achieving an average mapping rate of ~90%. Duplication rates ranged from 25%–35%, consistent with high library complexity and minimal technical (PCR) bias; low duplication rates are generally indicative of high‐quality RNA‐seq data. RNA‐seq differential expression analysis was performed using the DESeq2 package with default parameters [28]. The Wald test was used to compare the beta estimate divided by its estimated standard error to a standard normal distribution to derive *p*‐values. *p*‐values were then adjusted for multiple testing using the Benjamini and Hochberg method to produce false discovery rate (FDR) values. An FDR ≤ 0.05 was considered statistically significant. Gene Set Enrichment Analysis (GSEA) software [29, 30], and the Database for Annotation, Visualization and Integrated Discovery (DAVID) [31, 32] were used to perform pathway enrichment analysis. ChIP‐X Enrichment Analysis 3 (ChEA3) was used to identify upstream transcription regulatory targets of differentially expressed genes [33].

### 2.5 Co‐Immunoprecipitation (Co‐IP) and Western Blot Analysis

Co‐immunoprecipitation was performed using iPSC‐derived forebrain organoids. Protein samples were collected using 500 μL of IP lysis buffer supplemented with 5 μL of protease inhibitor cocktail and incubated on ice for 30 min. Immunoprecipitation was carried out using anti‐CEBPB (abcam, catalog #: ab32358, USA), or control IgG (Cell Signaling Technology, Danvers, MA, USA). Magnetic protein G beads (ThermoFisher, Dynabeads Protein G, catalog number: 10007D, USA) were added to the samples, which were rotated for 2 h at 4°C. Immunoprecipitates were washed three times with ice‐cold lysis buffer. CEBPB was immunoprecipitated using a CEBPB‐specific antibody, with IgG serving as a negative control to assess non‐specific binding. Approximately 1% of the input was loaded. The immunoprecipitated products were separated by SDS‐PAGE gel and immunoblotted for XBP‐1 s, TRIB3, and CEBPB. A light‐chain–specific secondary antibody was used to avoid interference from antibody heavy and light chains. Bound proteins were eluted using 50 μL of 4X Laemmli loading buffer and boiled at 95°C for 10 min. Proteins were then resolved on 4%–20% SDS‐PAGE gels and transferred to polyvinylidene fluoride membranes. The blocking buffer consisted of 5% skim milk in PBS. Membranes were incubated in blocking buffer for 1 h at room temperature with gentle agitation. After blocking, membranes were incubated overnight at 4°C with primary antibodies against CEBPB, TRIB3 or X‐box binding protein 1 (XBP1‐s). Membranes were washed with TBST for 5 min on the agitator five times. Membranes were then incubated with Rabbit TrueBlot ULTRA: Anti‐Rabbit IgG HRP—18‐8816‐31 for 1 h at room temperature. Signal detection was performed using Clarity Western ECL Blotting Substrate (Bio‐Rad, USA) and visualized with the GelDoc Go imaging system (Bio‐Rad, USA). A complete list of antibodies used for co‐IP and Western blotting is provided in Table S1.

### 2.6 CEBPB Chromatin Immunoprecipitation (ChIP) Assay

ChIP assays were conducted on iPSC‐derived forebrain neurons using the Pierce Magnetic ChIP Kit (Thermo Fisher Scientific, catalog #26157). DNA‐CEBPB complexes were immunoprecipitated with anti‐CEBPB antibodies, while normal rabbit IgG served as a negative control. Following purification, the recovered DNA was analyzed by qPCR using the primer sets listed in Table S1. ChIP enrichment was calculated as the percentage of ChIP DNA relative to input DNA (% input). To correct for variability in chromatin preparation, Ct values from each IP fraction were normalized to the corresponding input Ct values. Data are presented as percent input.

### 2.7 Statistical Analysis

Sequencing analyses were performed using R Statistical Software (version 4.5.0; R Foundation for Statistical Computing, Vienna, Austria). Figure 5 was analyzed using one‐way ANOVA (GraphPad Prism 10). *p* < 0.05 was considered statistically significant.

## 3 Results

### 3.1 Transcriptomic Profiles in iPSC‐Derived Forebrain Organoids

We generated iPSC‐derived forebrain organoids from three individuals, and all iPSC lines used in this study had normal karyotypes as determined by G‐band karyotyping analysis (Figure 1A). All iPSC lines expressed pluripotency markers, including SRY‐box transcription factor 2 (SOX2) and podocalyxin (TRA‐1‐81) (Figure 1B). We then cultured iPSC‐derived forebrain organoids in bioreactors (Figure 1A). Brain organoids were treated with fentanyl, liraglutide, or exenatide for 7 days (Figure 1C,D). RNA‐seq data demonstrated that the gene most strongly affected by fentanyl was TRIB3 (Figure 2A), which is a putative protein kinase that is induced by NF‐κB and associated with type 2 diabetes [34, 35]. TRIB3 is a regulator of the integrated stress response and modulated DDIT3‐dependent cell death during ER stress in neuronal cells [36, 37]. However, previous studies suggested that it did not affect non‐neuronal cells [36, 37]. Notably, TRIB3 was also significantly downregulated by both liraglutide and exenatide (Figure 2A). We subsequently explored genes involved in the ER stress response, including DNA damage‐inducible transcript 3 (DDIT3), activating transcription factor 4 (ATF4), protein phosphatase 1 regulatory subunit 15A (PPP1R15A), also known as growth arrest and DNA damage‐inducible protein (GADD34), and hexokinase domain containing 1 (HKDC1), all of which were downregulated in response to fentanyl, liraglutide, and exenatide (Figure 2A and Table S2). The ER stress‐related pathway was downregulated in iPSC‐derived forebrain organoids treated with fentanyl or GLP‐1 receptor agonists (Tables S3–S5). These findings indicate that ER stress modulation may represent a common transcriptional response to fentanyl and GLP‐1 receptor agonists. Therefore, we set out to determine the underlying mechanisms.

**FIGURE 2:**
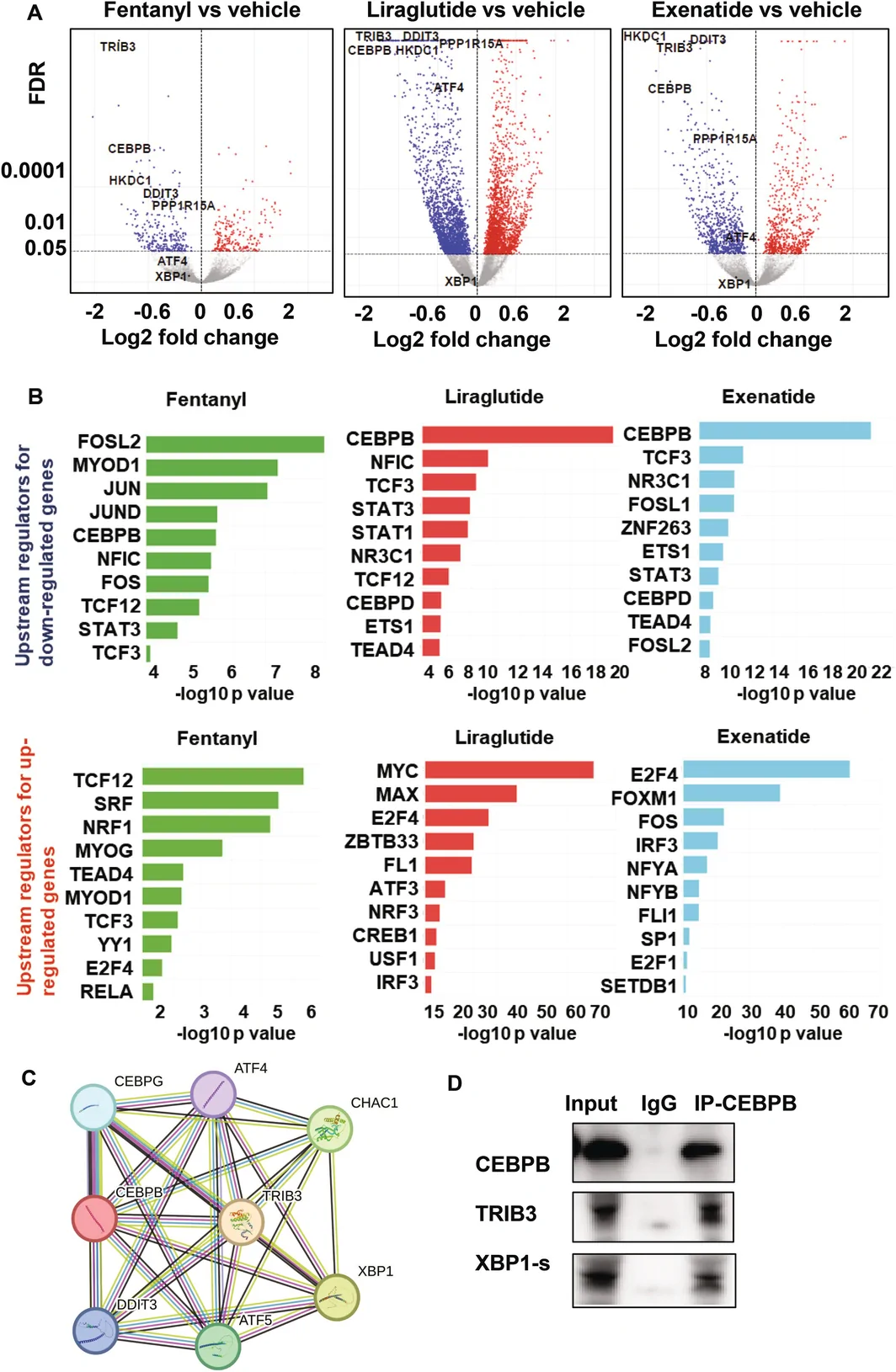
RNA‐seq analysis of iPSC‐derived forebrain organoids treated with fentanyl, liraglutide, and exenatide. (A) Volcano plots depict the most significantly altered gene expression profiles following drug treatment, based on RNA‐seq analysis (drug treatment vs. vehicle, FDR < 0.05). iPSC‐derived forebrain organoids from three individuals were included in the analysis. Each treatment condition was analyzed using two technical RNA‐seq replicates generated from independent library preparations. (B) Upstream regulator analysis was conducted using differentially expressed genes (FDR < 0.05). The top panel shows predicted upstream regulators associated with downregulated genes, whereas the bottom panel displays those linked to upregulated genes. (C) CEBPB emerged as the top upstream regulator of downregulated genes across all three drug‐treatment conditions. Analysis using the STRING database indicated that CEBPB interacts with multiple proteins involved in ER stress signaling, including TRIB3 and XBP1. (D) Co‐immunoprecipitation experiments confirmed a physical interaction between CEBPB and the spliced form of XBP1 (XBP1‐s) and TRIB3 in iPSC‐derived forebrain organoids.

### 3.2 CEBPB as an Upstream Regulator of ER Stress Genes

We identified CEBPB as the most significant upstream regulator of downregulated genes across all three treatment conditions (Figure 2B). Based on the STRING database (Figure 2C) [38], CEBPB can physically interact with TRIB3 and several ER stress‐related genes. We confirmed that CEBPB proteins interact with TRIB3 and XBP1s by co‐immunoprecipitation (Figure 2D). These findings suggest that ER stress regulation may represent a shared molecular response across fentanyl and GLP‐1 receptor agonists. Brain organoids contain various brain cell types, including neuronal and glial cells. As previously noted, TRIB3 regulates DDIT3‐dependent cell death in neuronal cells under ER stress [36, 37]. We then generated iPSC‐derived forebrain neurons for follow‐up mechanistic experiments.

### 3.3 Transcriptomic Profiles in iPSC‐Derived Forebrain Neurons

We hypothesized that modulation of ER stress may represent a common molecular mechanism underlying the therapeutic effects of drugs for SUDs. To test this hypothesis, we treated iPSC‐derived forebrain neurons with fentanyl, liraglutide, and exenatide, along with additional compounds, including tirzepatide, topiramate, gabapentin, and β‐hydroxybutyrate, all of which have shown therapeutic potential in the context of SUDs. Specifically, tirzepatide is a dual incretin receptor agonist that targets both GLP‐1 and GIP receptors, which may have a protective role in both OUD and alcohol use disorder (AUD) [10, 39]. Topiramate and gabapentin are anticonvulsants with demonstrated therapeutic potential for treating opioid withdrawal [40, 41]. β‐hydroxybutyrate, a ketone body, was included because of emerging evidence suggesting that ketogenic diets may benefit some individuals with SUDs, such as OUD and AUD [42, 43, 44]. Therefore, we generated iPSC‐derived forebrain neurons and treated them with fentanyl (3 nM) [20], liraglutide (50 nM) [21], exenatide (150 pM), tirzepatide (100 nM) [23], topiramate (5 μM) [24], gabapentin (22 μM) [25] or β‐hydroxybutyrate (5 mM) [26] (Figures 3A–3B). The selected drug concentrations fell within clinically relevant ranges and were informed by prior pharmacokinetic studies. Exenatide was removed from the subsequent analysis due to library preparation failure. Remarkably, several ER stress genes, including XBP1, TRIB3, and DDIT3, were downregulated (Figure 3B). XBP1 was one of the top hits across all treatment conditions. Normally, unspliced XBP1 mRNA is cleaved by the activated stress sensor IRE1α to generate spliced XBP1, a crucial transcription factor modulating ER stress response. Pathway enrichment analyses further demonstrated that downregulated genes were most strongly enriched for ER stress‐related pathways, including the unfolded protein response and IRE1α‐mediated chaperone activation (Figure S1). We performed additional Western blot analyses in iPSC‐derived neurons under ER stress conditions. Specifically, we examined key ER stress‐related proteins, including XBP1, PERK, ATF6, IRE1α, and BiP/GRP78. Our results demonstrated that thapsigargin‐induced ER stress was attenuated by treatment with fentanyl, liraglutide, tirzepatide, topiramate, gabapentin, and β‐hydroxybutyrate (Figure S2). In contrast, upregulated genes were linked to a broad range of pathways, including synaptic function, voltage‐gated channel activity, and neurotransmission (Figure S3). We performed a focused gene‐set enrichment analysis of six predefined Reactome pathways representing the global unfolded protein response (UPR) and its IRE1α/XBP1, PERK–eIF2α–ATF4, and ATF6 branches. We found negative normalized enrichment scores across the six drug treatment comparisons. Specifically, the global UPR and both ATF6‐related gene sets were significantly negatively enriched in all six comparisons, with FDR < 0.05. In contrast, thapsigargin produced significant positive enrichment of all six ER stress‐related pathways, supporting the expected directionality of the analysis (Figure 3C). Collectively, these findings identify modulation of the ER stress response as a shared transcriptional feature of these diverse treatment conditions.

**FIGURE 3:**
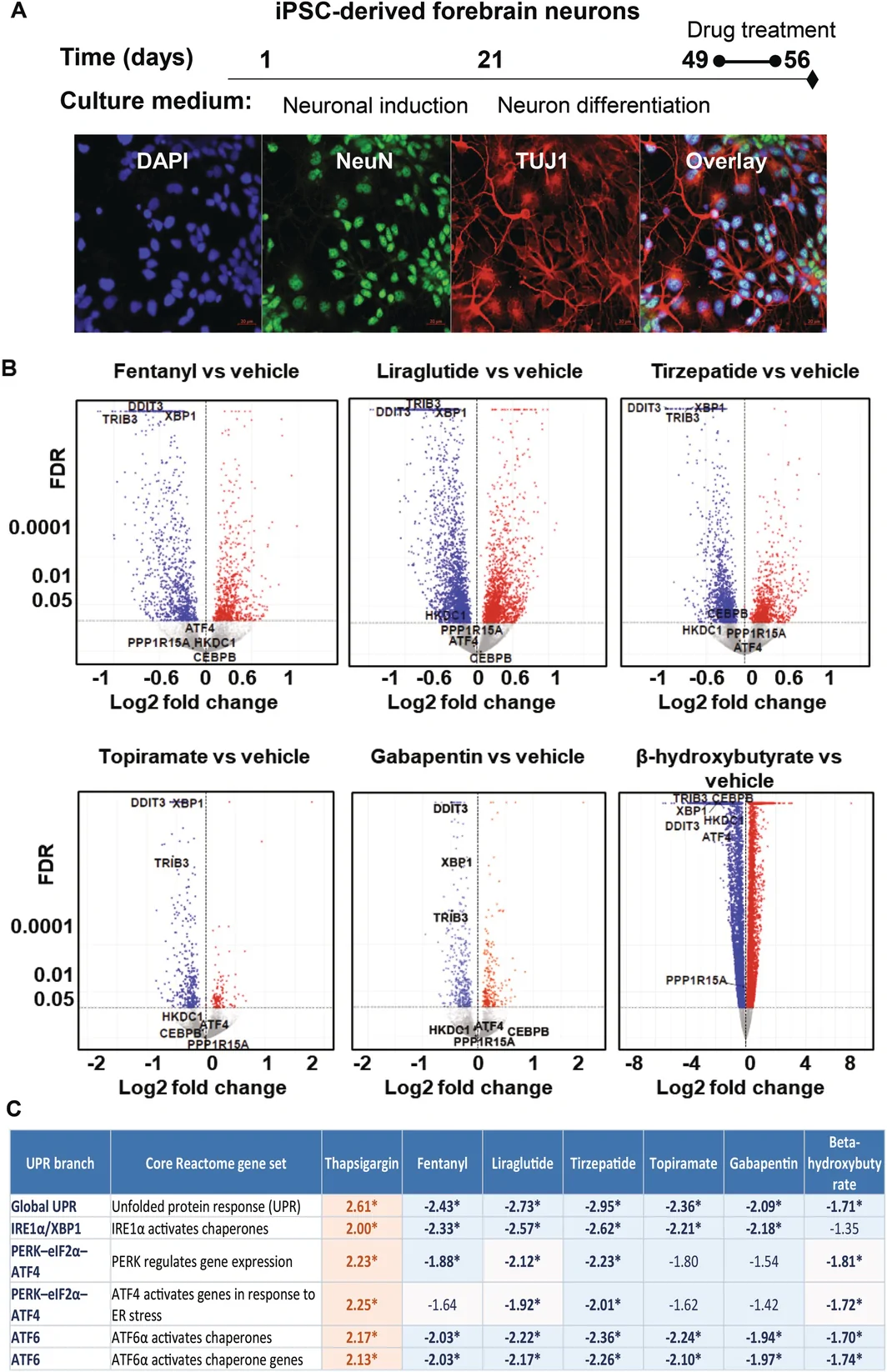
RNA‐seq analysis of iPSC‐derived forebrain neurons treated with fentanyl, liraglutide, tirzepatide, topiramate, gabapentin, and β‐hydroxybutyrate. (A) A schematic overview of the differentiation protocol used to generate the iPSC‐derived forebrain neurons. (B) Volcano plots depict the most significantly altered gene expression profiles following drug treatment, based on RNA‐seq analysis (drug treatment vs. vehicle, FDR < 0.05). iPSC‐derived forebrain neurons from a single individual were used for this RNA‐seq analysis. Each treatment condition was analyzed with three technical replicates generated from independent library preparations. (C) Focused GSEA identified a coherent, branch‐spanning ER‐stress/UPR signature. The comparison showed positive enrichment for all six core gene sets, each at FDR *q* < 0.05. Because several Reactome gene sets are nested, these results are presented as convergent branch‐level evidence rather than as independent pathway discoveries. Conversely, across the six drug comparisons, all NES values for the core ER stress pathway comparisons were negative. The global UPR and both ATF6 gene sets were significant in all six comparisons. *FDR *q* < 0.05.

### 3.4 Drug Treatment Reduces CEBPB Binding at ER Stress‐Related Genes

We identified 158 differentially expressed genes (DEGs) that overlapped with all treatment conditions, the majority of which were downregulated (Figure 4A). Strikingly, CEBPB again emerged as the top upstream regulator of these DEGs (FDR = 0.00168), with 17 genes predicted as CEBPB targets (Figure 4B). Even more striking, many of these targets were associated with ER stress, including DDIT3, TRIB3, XBP1, heat shock protein family A (Hsp70) member 9 (HSPA9), solute carrier family 7 member 11 (SLC7A11), fibronectin 1 (FN1), fin bud initiation factor homolog (FIBIN), and homocysteine inducible ER protein with ubiquitin like domain 1 (HERPUD1) (Figure 4B). We further validated these findings using our iPSC‐derived cell model system. Specifically, iPSC‐derived neurons were treated with thapsigargin, a pharmacological inducer of ER stress. As anticipated, DDIT3, TRIB3, HSPA9, XBP1, SLC7A11, FN1, FIBIN, and HERPUD1 mRNA levels were upregulated, and the ER stress response emerged as the most significantly affected pathway (Figure 4C and Table S6).

**FIGURE 4:**
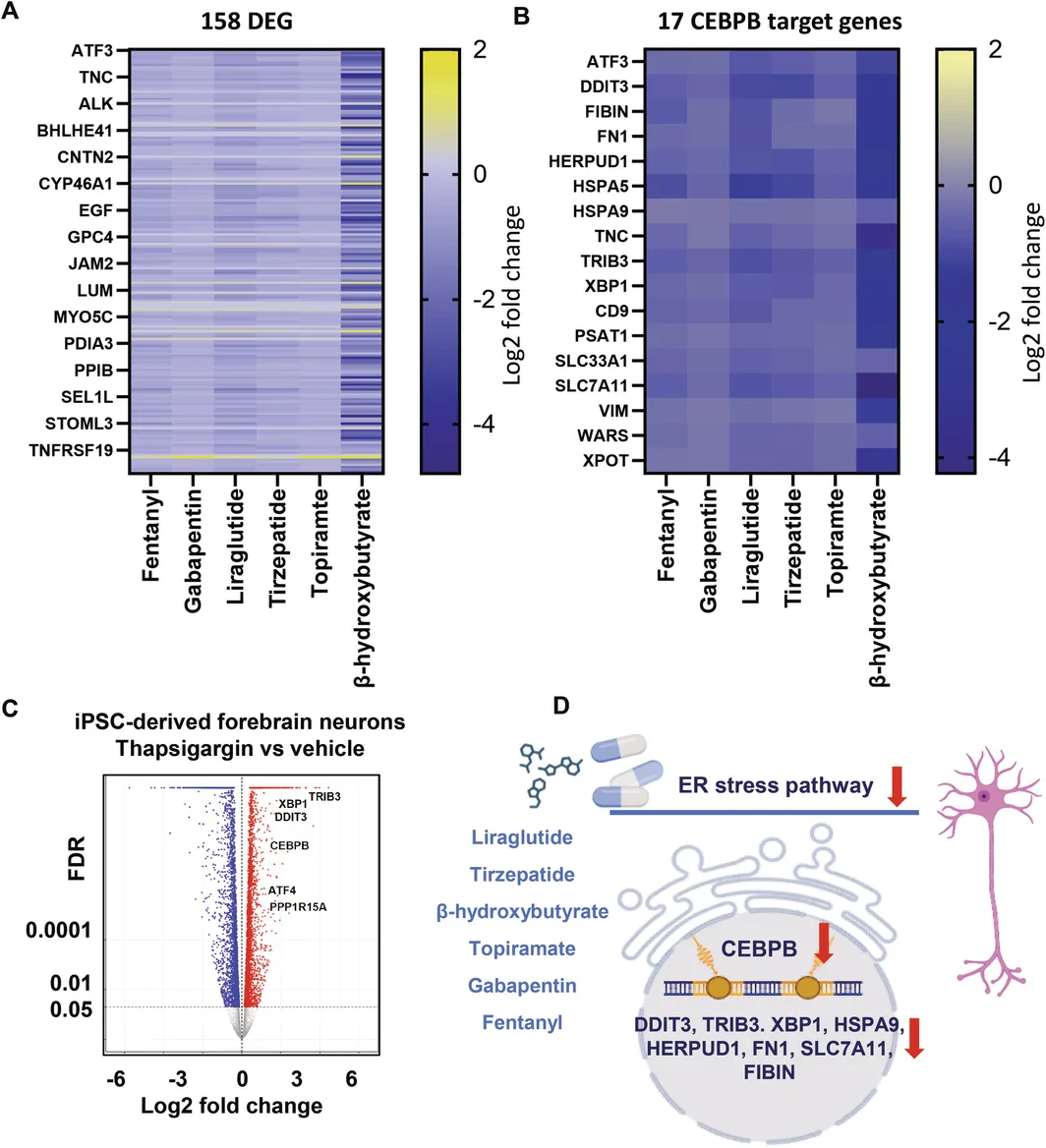
CEBPB DNA‐binding activity may contribute to modulation of the ER stress signaling pathway in response to drug treatment. (A) A total of 158 differentially expressed genes (DEGs) overlapped across all treatment conditions, with most showing downregulation (FDR < 0.05). (B) Of these 158 DEGs, 17 were identified as CEBPB target genes using the ChEA3 database. All 17 targets were downregulated following drug treatment (FDR < 0.05). (C) Volcano plots depict the most significantly altered gene expression profiles following drug treatment, based on RNA‐seq analysis (thapsigargin vs. vehicle, FDR < 0.05). iPSC‐derived forebrain neurons from a single individual were used for this RNA‐seq analysis. Each treatment condition was analyzed with three technical replicates generated from independent library preparations. (D) The proposed study model demonstrates that these drug treatments may alter CEBPB DNA‐binding activity, thereby affecting downstream gene regulation involved in ER stress signaling.

Although CEBPB acts as a transcription factor, its mRNA levels remained unchanged following treatment with fentanyl, liraglutide, tirzepatide, topiramate, gabapentin, or β‐hydroxybutyrate in iPSC‐derived forebrain neurons. We hypothesized that these treatments may modulate CEBPB's DNA‐binding activity rather than its expression, thereby influencing downstream gene regulation (Figure 4D). To test this hypothesis, we first queried the Encyclopedia of DNA Elements (ENCODE) database and identified CEBPB binding sites in several of the above‐mentioned ER stress‐related genes, with strong signal intensities ranging from 500 to 1000. This prompted us to perform CEBPB ChIP assays to validate these interactions. As anticipated, CEBPB could bind to most of those ER stress‐related genes in the absence of drug treatment (Figure 5). Remarkably, CEBPB DNA binding was significantly decreased in response to drug treatments (Figure 5). Together, these findings indicate that diverse SUD‐relevant compounds produced a shared ER stress‐related transcriptional response, potentially associated with reduced CEBPB binding to its target genes.

**FIGURE 5:**
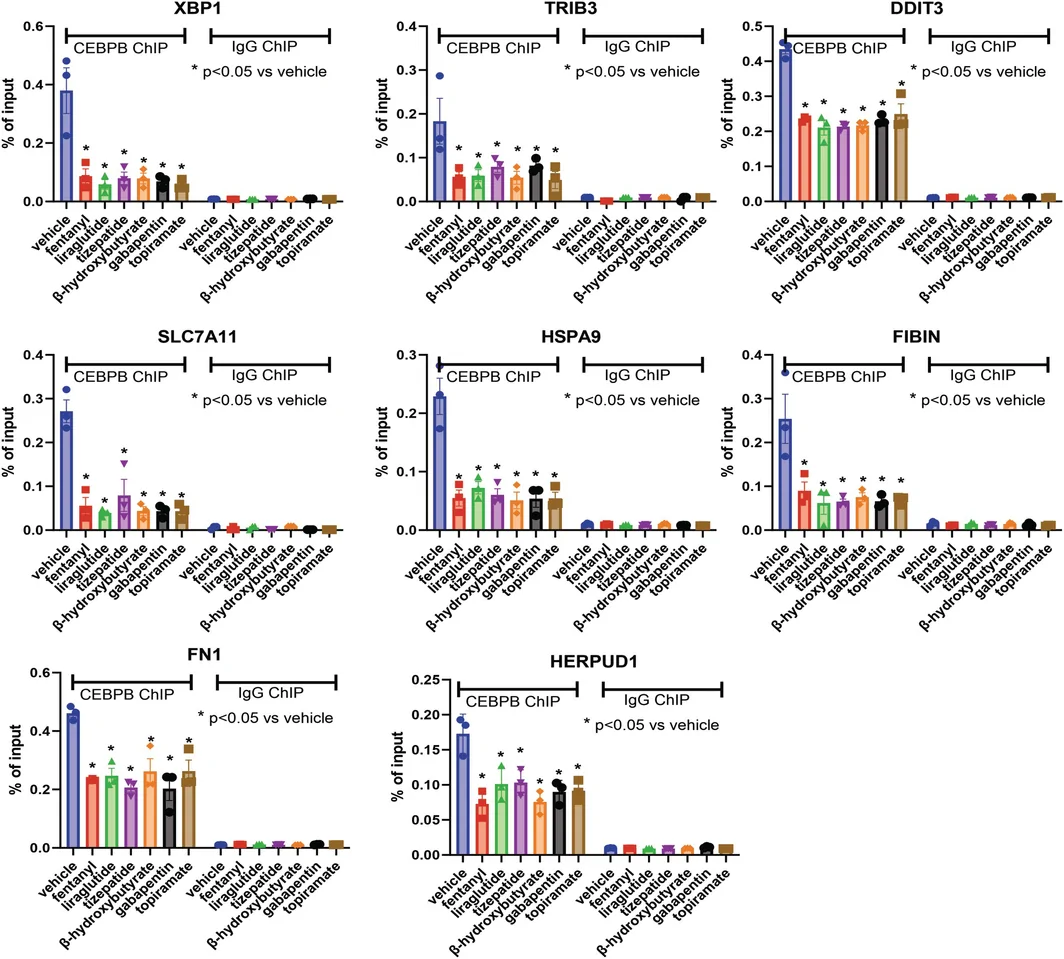
CEBPB‐driven ER stress attenuation unifies the actions of fentanyl, GLP‐1 receptor agonists, and candidate SUD therapeutics. ChIP assays were conducted in iPSC‐derived forebrain neurons, confirming that changes in CEBPB binding correlated with altered mRNA expression of eight ER stress‐related genes. ChIP DNA enrichment was quantified by qPCR and expressed as % input (*n* = 3). ChIP‐qPCR was performed in triplicate. Drug treatments were compared with vehicle controls using one‐way ANOVA followed by Tukey–Kramer post hoc analysis. CEBPB ChIP and IgG control samples were analyzed separately. *p < 0.05 vs. vehicle. Three independent experiments were performed.

## 4 Discussion

Our findings demonstrate attenuation of ER stress signaling as a convergent transcriptional feature across fentanyl, GLP‐1 receptor agonists, anticonvulsants, and metabolic modulators in human iPSC‐derived neural models. Across these pharmacologically distinct drug classes, ER stress‐related genes were consistently suppressed, with CEBPB emerging as a common upstream transcriptional regulator. These results position ER stress modulation as a candidate link among these treatment‐associated responses and suggest potential strategies for the development or repurposing of treatments for OUD.

Fentanyl and GLP‐1 receptor agonists, despite acting on pharmacologically distinct primary targets, produced overlapping transcriptional changes involving attenuation of ER stress signaling in human iPSC‐derived forebrain organoids. In the present study, the fentanyl concentration used was 3 nM, which is substantially lower than concentrations commonly used in most in vivo and in vitro studies (typically ranging from 100 nM to 250 μM) [45]. The recommended serum concentrations of fentanyl for analgesia and anesthesia are 1–2 ng/mL (~2.97–5.94 nM) and 10–20 ng/mL (~29.7–59.4 nM), respectively, with a lethal dose of fentanyl in humans estimated at 2 mg. Blood concentrations approximately 7 ng/mL or higher have been linked to deaths in cases involving polysubstance misuse. We emphasize that drug effects can vary markedly depending on treatment conditions, including dose, duration, and frequency of exposure. The concentration used here was selected to be clinically relevant based on human pharmacokinetic studies.

Liraglutide is a 31‐amino acid polypeptide chain with the molecular formula C₁₇_2_H_2_₆₅N₄₃O₅₁ and a molecular weight of 3751.26 Da. Exenatide is a 39‐amino acid synthetic peptide amide with the molecular formula C₁₈₄H_2_₈_2_N₅₀O₆₀S and a molecular weight of 4186.6 Da. Although these two GLP‐1 receptor agonists belong to the same drug class, we observed a greater number of differentially expressed genes in iPSC‐derived forebrain organoids treated with exenatide (Figure 2A). This observation aligns with our previous study, in which oxycodone and buprenorphine produced distinct transcriptional profiles in iPSC‐derived forebrain organoids despite both targeting opioid receptors [15]. These findings indicate that individual drugs within the same class can produce unique molecular signatures, underscoring the importance of patient‐derived models for evaluating drug effects in the human brain. It should be noted that several immune‐related pathways were downregulated across all three treatments (Tables S3–S5). Both drug classes produced similar transcriptional signatures in genes involved in ER stress. ATF4 represents an additional point of convergence between transcriptomic and protein network analyses. ATF4 is a principal downstream effector of the PERK–eIF2α arm of the integrated stress response and regulates several genes identified in this study, including DDIT3, TRIB3, and PPP1R15A. The coordinated downregulation of ATF4 and these downstream targets, together with enrichment of the Reactome “ATF4 activates genes in response to endoplasmic reticulum stress” gene set, is consistent with attenuation of ATF4‐associated transcriptional signaling. However, ATF4 protein abundance, eIF2α phosphorylation, and ATF4 DNA‐binding activity were not directly measured. Thus, these results do not establish direct inhibition of the PERK–eIF2α–ATF4 pathway.

The most significant suppression of TRIB3 was observed after fentanyl exposure, and its expression was similarly reduced by liraglutide and exenatide in iPSC‐derived forebrain organoids (Figure 2A). In line with this observation, TRIB3 mRNA expression was consistently downregulated across all treatment conditions in iPSC‐derived forebrain neurons (Figure 3B). Importantly, the drug concentrations used in this study were selected to approximate clinically relevant exposure, supporting further evaluation of ER stress modulation as a potential shared response to fentanyl and GLP‐1 receptor agonists. We identified CEBPB as a candidate transcriptional regulator associated with these shared responses.

CEBPB plays an important role in ER stress, inflammation, and metabolic disturbances [46]. A key insight from this work is the identification of CEBPB as a common upstream regulator whose activity, rather than expression, appears to be modulated by these compounds in iPSC‐derived forebrain neurons. Although CEBPB mRNA levels remained unchanged, its predicted downstream targets, including numerous ER stress genes, were consistently downregulated. ChIP assays confirmed direct CEBPB binding to multiple ER stress‐related loci under basal conditions, and drug treatments significantly reduced this DNA‐binding activity. These findings suggest that CEBPB acts as a regulatory hub through which pharmacologic interventions converge to attenuate ER stress signaling. Given previous reports linking CEBPB activity to cellular stress responses, neuroinflammation, and synaptic plasticity, diminished DNA binding of CEBPB may represent a crucial mechanism through which drugs exert neuroprotective effects [47]. Although the coordinated transcriptional, protein, and CEBPB‐binding results support modulation of ER‐stress‐associated signaling, global protein synthesis and neuronal activity were not measured concurrently in these experiments. We therefore cannot exclude the possibility that some of the observed changes are secondary to altered neuronal activity or translation. Future studies integrating measurements of neuronal activity, nascent protein synthesis, and ER‐stress signaling will be required to establish the temporal and causal relationships among these processes.

Several anti‐diabetic medications, including metformin and liraglutide, have been shown to exert indirect ER stress‐modulating effects [48]. The convergence of fentanyl and GLP‐1 receptor agonists in their effects on ER stress‐related pathways carries important implications for understanding therapeutic action in SUDs. Extending this analysis to additional compounds, including anticonvulsants and metabolic modulators, further supports the concept that therapeutic effects in SUDs may depend not only on receptor‐specific effects but also on the restoration of cellular homeostasis via attenuation of ER stress. ER stress signaling is increasingly recognized as a critical contributor to addiction biology, as chronic drug exposure (e.g., opioids) activates cellular stress pathways that drive neuroplastic changes across mesocorticolimbic circuits [49]. ER stress influences synaptic remodeling, neuroinflammation, and vulnerability to neurotoxicity, processes that may influence reward learning and behavioral sensitization [50]. In this context, the convergence of fentanyl and GLP‐1 receptor agonists on the downregulation of ER stress‐associated genes suggests that attenuation of ER stress may represent a shared protective or homeostatic mechanism that counteracts maladaptive plasticity induced by addictive substances. Recent real‐world data suggest that GLP‐1 agonists may reduce drug‐seeking behaviors and alter reward circuitry through mechanisms beyond their primary receptor‐specific actions [51, 52]. The convergence of GLP‐1 receptor agonists, anticonvulsants, metabolic modulators, and opioid ligands on a shared ER stress signature underscores the possibility that therapeutic efficacy in SUDs may depend not only on receptor‐specific mechanisms but also on broader restoration of cellular homeostasis. Our results may help explain why certain drugs with distinct primary targets nonetheless show overlapping clinical benefits in treating opioid withdrawal, AUD, and other SUD‐related conditions. While further work is needed to determine whether pharmacologic modulation of ER stress can be leveraged therapeutically for SUDs, our results point to a promising avenue for drug repurposing and mechanism‐guided treatment development.

Several limitations to this study should be noted. We initially conducted RNA‐seq on iPSC‐derived forebrain organoids, but the small sample size limits the broader applicability of our findings. Our iPSC‐derived brain‐like cells are region‐specific. We investigated iPSC‐derived forebrain organoids, a part of the brain that has been implicated in the pathophysiology of addiction [53, 54]. Given the complexity of the human brain, focusing on a single region may not fully capture the intricacies of the entire central nervous system. However, the iPS cell model system, particularly in psychiatric research, represents a powerful research tool for generating and testing mechanistic hypotheses. Future research should expand to include additional brain regions and leverage single‐cell sequencing in iPSC‐derived brain organoids to explore cell‐type‐specific and drug‐specific effects more comprehensively. Despite these limitations, our study provides novel mechanistic insights into drug actions in iPSC‐derived brain‐like cells. The ER stress signature observed across treatments and platforms is consistent with a convergent transcriptional response. Further work will be needed to determine whether modulating CEBPB‐dependent transcriptional networks yields therapeutic benefits and whether these ER stress‐related mechanisms extend to in vivo or clinical settings.

## 5 Conclusions

This study identifies a shared ER stress‐related transcriptional response to fentanyl, GLP‐1 receptor agonists, and several compounds being evaluated for SUD treatment. Fentanyl, liraglutide, and exenatide consistently downregulated ER stress‐related genes, with TRIB3 emerging as one of the most strongly affected genes across treatments in iPSC‐derived forebrain organoids. Additional stress‐response genes, including DDIT3, ATF4, PPP1R15A, and HKDC1, were similarly suppressed, indicating broad attenuation of ER stress signaling. We identified CEBPB as a candidate upstream regulator of these shared transcriptional changes. Functional studies in iPSC‐derived forebrain neurons further revealed downregulation of ER stress genes and reduced CEBPB occupancy across diverse drug classes, including anticonvulsants and metabolic modulators. Together, these findings highlight ER stress modulation, potentially involving CEBPB, as a convergent response that warrants further evaluation for SUDs.

## Author Contributions

M.‐F. Ho wrote the manuscript. M.‐F. Ho designed the research. M.‐F. Ho and C. Zhang performed the research. C. Zhang and H. Li analyzed the data and contributed analytical tools. All authors approved the final version of the manuscript.

## Funding

This work was supported in part by the National Institutes of Health [grant numbers K01 AA28050, R01 AA27486, R01 AA33316 and R01 DA57928]; the Brain & Behavior Research Foundation [grant number 31329], the Mayo Clinic Research Pipeline K2R Program, the Terrance and Bette Noble Foundation, and a generous gift from Donna Giordano.

## Ethics Statement

This study was conducted in accordance with the protocol #20–00372, which was reviewed and approved by the Mayo Clinic Institutional Review Board.

## Consent

Participant confidentiality was strictly maintained throughout the study. Written consent was obtained from all individuals whose samples were used, including consent for study participation and publication of the results in a peer‐reviewed scientific journal.

## Conflicts of Interest

The authors declare no conflicts of interest.

## Supporting information


**Figure S1:** Pathway enrichment analysis was performed using downregulated genes (drug treatment vs. vehicle, FDR < 0.05).
**Figure S2:** Representative western blot analysis to quantify ER stress‐related molecule levels in iPSC‐derived forebrain neurons. Cells were treated with thapsigargin (100 nM) to induce ER stress. Thapsigargin‐induced ER stress was attenuated by treatment with fentanyl, liraglutide, tirzepatide, topiramate, gabapentin, and β‐hydroxybutyrate.
**Figure S3:** Pathway enrichment analysis was performed using upregulated genes (drug treatment vs. vehicle, FDR < 0.05).

**Table S1:** Primer sequences and antibodies used for experiments.
**Table S2:** RNAseq results in iPSC‐derived forebrain organoids treated with fentanyl, exenatide, or liraglutide.
**Table S3:** Pathway analysis using RNA‐seq results in iPSC‐derived forebrain organoids treated with fentanyl.
**Table S4:** Pathway analysis using RNA‐seq results in iPSC‐derived forebrain organoids treated with exenatide.
**Table S5:** Pathway analysis using RNA‐seq results in iPSC‐derived forebrain organoids treated with liraglutide.
**Table S6:** RNA‐seq results in iPSC‐derived forebrain neurons treated with thapsigargin.

## Data Availability

All data supporting our findings are available in the main article or its supplementary files (Table S1–S6; Figure S1–S3). Sequencing data are available via the GEO database: GSE315126 and GSE315127.
