# Supplementary material for: Modulation of Endoplasmic Reticulum Stress via CEBPB: A Potential Molecular Link to Therapeutic Action in Substance Use Disorders
Source: CNS Neurosci Ther. 2026 Sep 29;32(10):e71183. doi: 10.1002/cns.71183 (PMC13621937; doi:10.1002/cns.71183)

Modulation of Endoplasmic Reticulum Stress via CEBPB: a potential molecular link to therapeutic action in substance use disorders

Cheng Zhang, Ph.D.[2], Hu Li, Ph.D.[2] , Ming-Fen Ho, Ph.D.[1,2*]

[1] Department of Psychiatry and Psychology, Mayo Clinic; Rochester, Minnesota, USA
[2] Department of Molecular Pharmacology and Experimental Therapeutics; Mayo Clinic, Rochester, Minnesota, USA

***Corresponding author**
Ming-Fen Ho, Ph.D., Mayo Clinic 200 First Street SW, Rochester, MN 55905,
Phone: 507-2293-3387, Email: ho.mingfen@mayo.edu

Supplementary Figure 1. Pathway enrichment analysis was performed using downregulated genes (drug treatment vs. vehicle, FDR < 0.05).

Supplementary Figure 2. Representative western blot analysis to quantify ER stress-related molecule levels in iPSC-derived forebrain neurons. Cells were treated with thapsigargin (100 nM) to induce ER stress. Thapsigargin-induced ER stress was attenuated by treatment with fentanyl, liraglutide, tirzepatide, topiramate, gabapentin, and β-hydroxybutyrate.

Supplementary Figure 3. Pathway enrichment analysis was performed using upregulated genes (drug treatment vs. vehicle, FDR < 0.05).

Supplementary Figure 1. Pathway enrichment analysis was performed using downregulated genes (drug treatment vs. vehicle, FDR < 0.05).

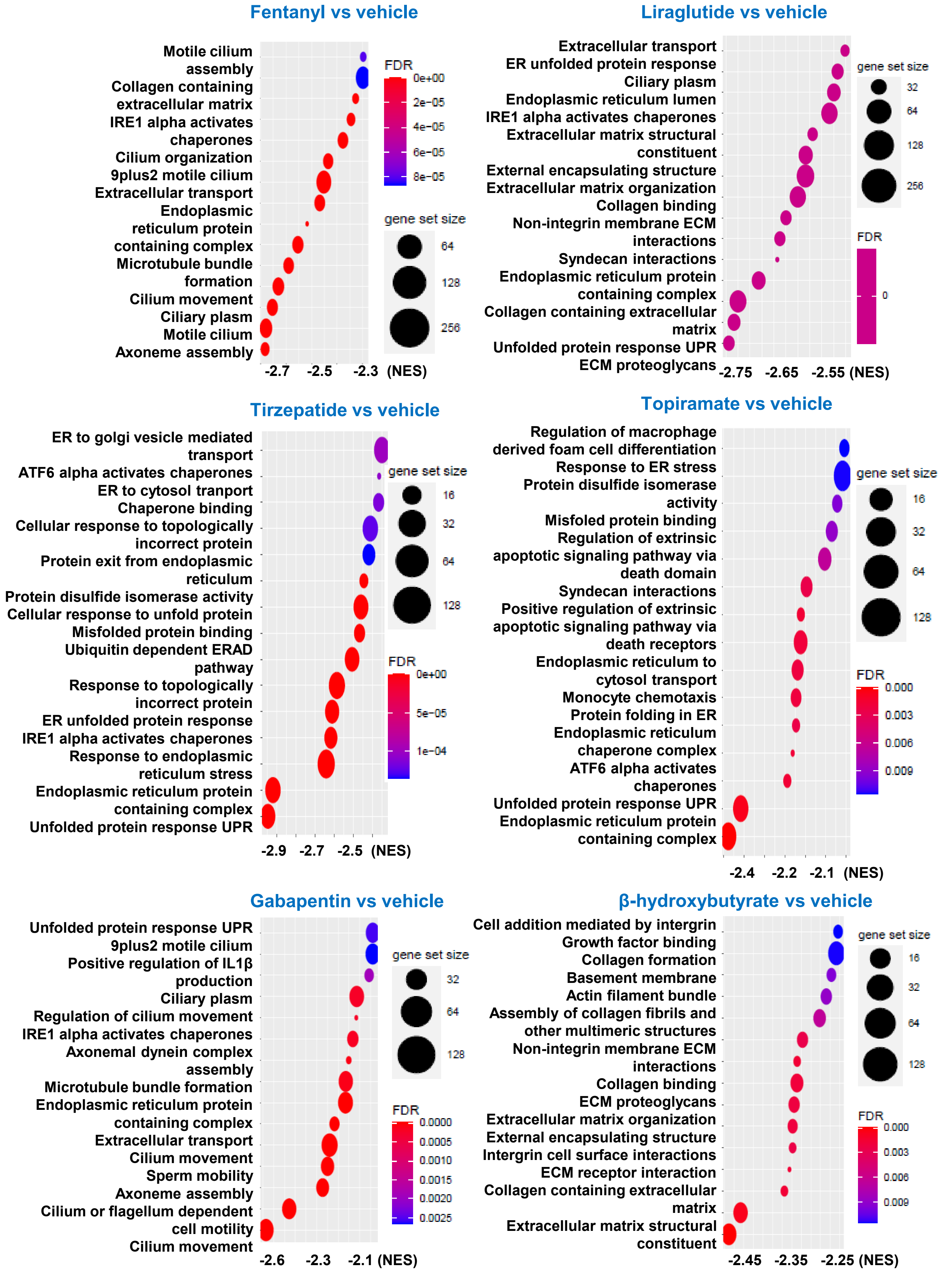

Supplementary Figure 2. Representative western blot analysis to quantify ER stress-related molecule levels in iPSC-derived forebrain neurons. Cells were treated with thapsigargin (100nM) to induce ER stress. Thapsigargin-induced ER stress was attenuated by treatment with liraglutide, tirzepatide, topiramate, gabapentin, and β-hydroxybutyrate

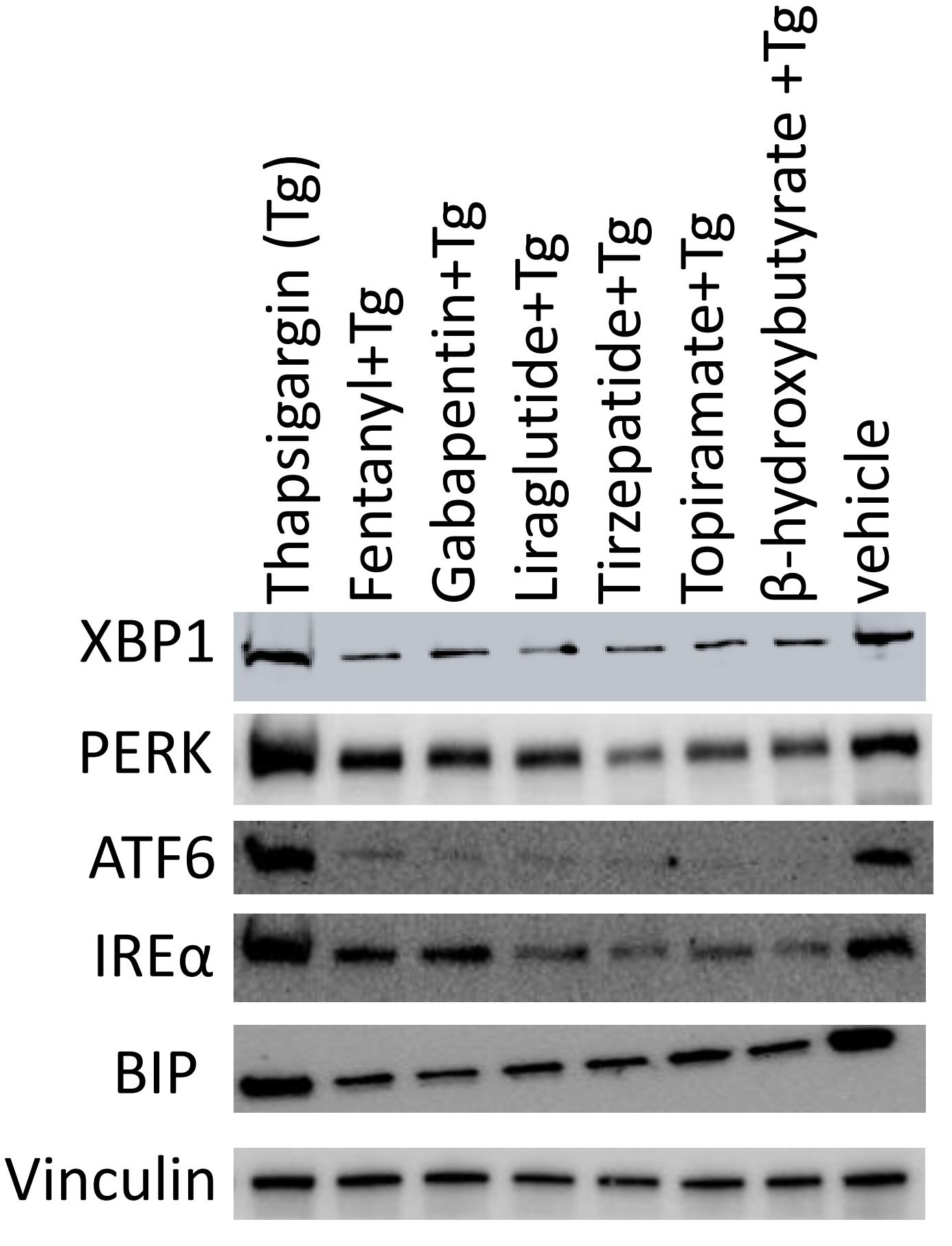

Supplementary Figure 3. Pathway enrichment analysis was performed using upregulated genes (drug treatment vs. vehicle, FDR < 0.05).

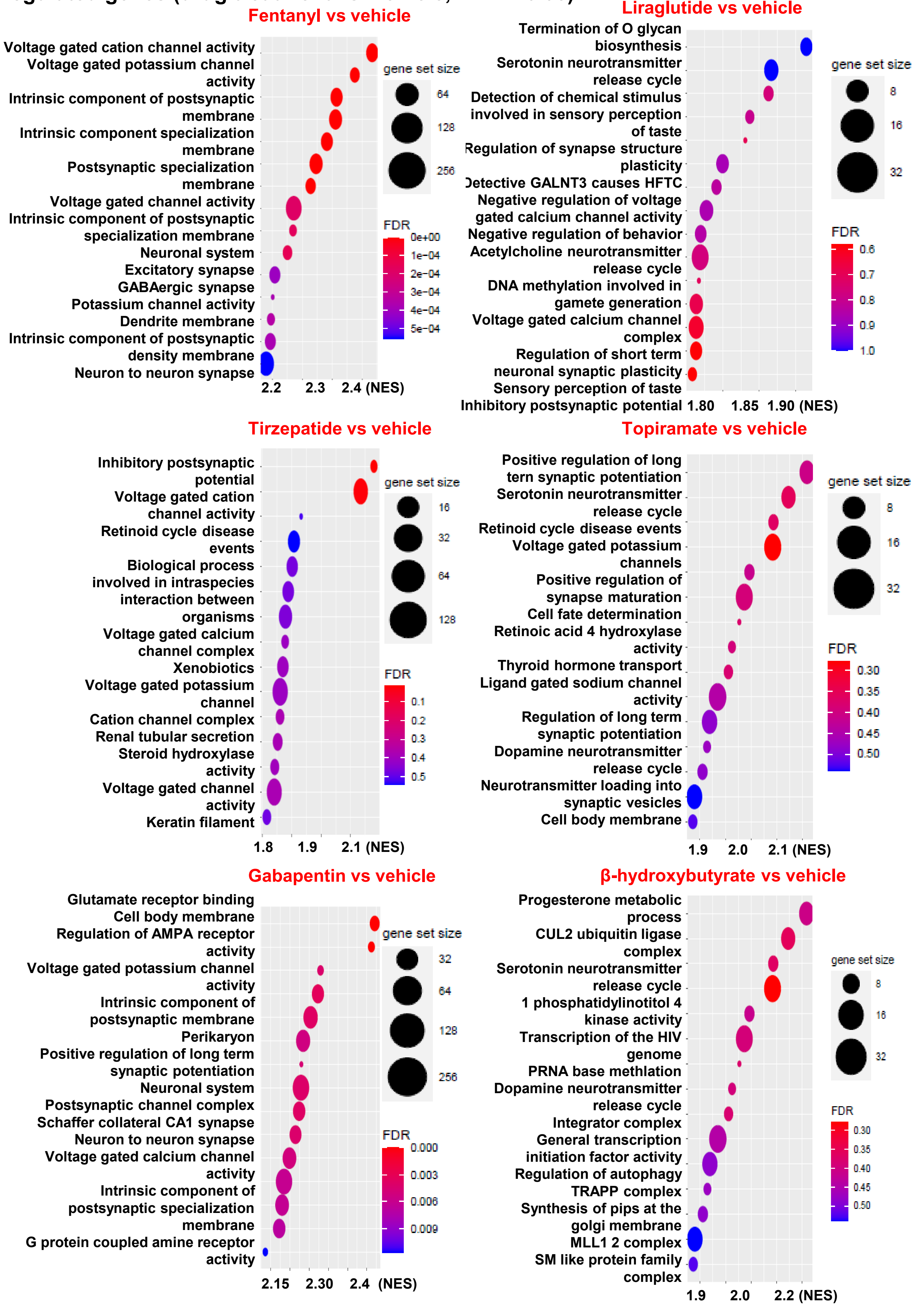

Supplement: Supplementary file 1 [file CNS-32-e71183-s001.pdf]
